# Authentic Leadership and Improved Individual Performance: Affective Commitment and Individual Creativity’s Sequential Mediation

**DOI:** 10.3389/fpsyg.2021.675749

**Published:** 2021-05-07

**Authors:** Ana Patrícia Duarte, Neuza Ribeiro, Ana Suzete Semedo, Daniel Roque Gomes

**Affiliations:** ^1^Business Research Unit, Instituto Universitário de Lisboa, Lisbon, Portugal; ^2^Center for Applied Research in Management, Instituto Politécnico de Leiria, Leiria, Portugal; ^3^School of Management, Hospitality and Tourism, University of the Algarve, Portimão, Portugal; ^4^Center for Business and Economics Research, University of Coimbra, Coimbra, Portugal; ^5^Instituto Politécnico de Coimbra, Escola Superior de Educação de Coimbra, Coimbra, Portugal; ^6^NOVA Institute of Communication, University NOVA, Lisbon, Portugal

**Keywords:** authentic leadership, affective commitment, individual creativity, individual performance, sequential mediation model

## Abstract

Authentic leadership has become increasingly important in the literature, attracting the attention of many scholars in the last decade. This study adopted an employee-centered perspective to guide its examination of the relationship between authentic leadership and individual performance and investigation of the sequential mediation of employees’ affective commitment and individual creativity. An analysis was conducted of data collected from 214 employees working in different business sectors. The results reveal a statistically significant positive relationship between authentic leadership and employees’ workplace performance, which are both directly connected and indirectly linked through the two proposed psychosocial mechanisms. The findings thus indicate that authentic leadership reinforces workers’ emotional connection with their organizations, thereby increasing their individual creativity and, subsequently, promoting better on-the-job performance. This study presents new and significant results since, on the one hand, it relied on a sequential mediation analysis of variables and, on the other hand, integrated the four main constructs into a single model. The proposed model displays the chain of effects between authentic leadership, affective commitment, individual creativity, and employee workplace performance. Implications for organizational management are discussed.

## Introduction

Authentic leadership has emerged as an important area of research in the field of organizational behavior in the past decade (Farid et al., 2020). This leadership style has been defined as a process that draws from both “positive psychological capacities and a highly developed organizational context, which results in both greater self-awareness and self-regulated positive behaviors on the part of leaders and associates, [thereby] fostering positive self-development” (Luthans and Avolio, 2003, p. 243). Walumbwa et al. (2008) identify four underlying dimensions of authentic leadership: self-awareness, relational transparency, balanced information processing, and an internalized moral perspective. Authentic leaders thus say exactly what they mean, act on their stated beliefs, seek out opinions that challenge their personal views, and ask for feedback to improve interpersonal interactions (Laschinger et al., 2012). As a result, these leaders reinforce employees’ positive attitudes and behaviors, generating benefits for specific workgroups and entire organizations.

More specifically, authentic leadership enhances employees’ individual performance (Clapp-Smith et al., 2009; Wang et al., 2014; Ribeiro et al., 2018a). Social exchange theory (Blau, 1964) contributes to explaining why employees who perceive an authentic leadership style in their supervisors develop a feeling of obligation to reciprocate with improved performance. Previous research has also indicated that authentic leadership elevates workers’ performance by promoting employees’ affective commitment and individual creativity (Ribeiro et al., 2018b). Affective commitment refers to workers’ emotional attachment to their organization (Meyer and Allen, 1991). The literature shows that, of the three components of organizational commitment (i.e., affective, normative, and instrumental), the affective dimension has more desirable implications for individuals’ behavior within organizations. Leaders’ authenticity can influence employees’ emotional attachment to their organization, so authentic leaders may also inspire feelings of affective commitment in employees (Zhou et al., 2014; Alshammari et al., 2015; Milić et al., 2017; Semedo et al., 2017, 2018, 2019; Ribeiro et al., 2018a, b, 2020.

Authentic leadership can further positively influence followers’ workplace creativity (Müceldili et al., 2013; Xu et al., 2017; Ribeiro et al., 2018a), which can be understood as the production of new useful ideas and solutions (Amabile, 1988; Binnewies et al., 2008). Although creativity can be considered both an outcome and a process (Shalley and Zhou, 2008), the present study treated individual creativity as an outcome, that is, the extent to which employees produce fresh ideas and valuable solutions. Leaders with personal moral standards who are transparent in their actions and interactions with others are perceived by workers as authentic leaders who create a positive, appealing, and supportive organizational environment (Madjar et al., 2002), which makes followers more prone to creative behaviors. Authentic leaders also increase employees’ positive emotions, thereby reinforcing individual creativity (Leroy et al., 2012; Černe et al., 2013; Banks et al., 2016). Positive emotions specifically increase workers’ desire to explore and assimilate new ideas, discover fresh information, and develop their individual potential, which induces employees to be more creative (Wright and Cropanzano, 2004).

The current research’s results support the assertion that leadership strongly influences employee performance. Previous studies have also highlighted the authentic leadership style as a significant determinant of individual performance (Wang et al., 2014; Ribeiro et al., 2018a). Researchers have further thoroughly documented authentic leadership’s close connection with affective commitment (Milić et al., 2017; Semedo et al., 2017, 2018, 2019; Ribeiro et al., 2018a, b, 2020) and individual creativity (Müceldili et al., 2013; Xu et al., 2017; Ribeiro et al., 2018a). However, the existing literature provides no clear evidence of studies that have examined affective commitment and individual creativity’s sequential mediation in the relationship between authentic leadership and individual performance. This research is, therefore, the first to propose that authentic leadership influences employees’ individual performance both directly and indirectly through two sequential mediators: affective commitment and individual creativity.

## Research Background and Hypotheses Development

### Authentic Leadership and Individual Performance

Authentic leaders’ behavior is strongly rooted in beliefs, values, and moral principles that stimulate workers’ performance (Alshammari et al., 2015). According to Levy (2020), the relationship between authentic leaders’ ethical behavior and employees’ improved performance can be understood as that the latter feel authorized to act more freely within the parameters of their jobs and that this self-sufficiency influences performance.

Researchers have previously found authentic leadership to be positively related to employee performance (Clapp-Smith et al., 2009; Wang et al., 2014; Ribeiro et al., 2018a). The social support and social learning theories (Bandura, 1977) both suggest that, when employees sense their leaders’ respect, consideration, concern, and support and perceive them as authentic, workers can more easily excel (Hinojosa et al., 2014). Social exchange theory (Blau, 1964) can also be used to explain why individuals who perceive authentic leadership develop a feeling of obligation to reciprocate with improved performance. The latter theory proposes that each person’s behavior is contingent on other individuals’ behavior. Subordinates working under an authentic leader’s guidance may feel, in accordance with the norm of reciprocity (Gouldner, 1960), the need to improve their performance to repay the leader’s positive behaviors and thus balance the exchange relationship (Cropanzano and Mitchell, 2005). Prior studies have confirmed that followers respond to their leaders’ authenticity by performing better as individuals (e.g., Wang et al., 2014; Ribeiro et al., 2018a).

In addition, the broaden-and-build theory (Fredrickson, 2004) posits that, when staff members are exposed to positive emotions, these feelings will have long-term effects on workers that are essential for successful individual performance. Hao et al. (2020) found that individuals experiencing upbeat emotions and deep trust repay their leaders with higher performance. Thus, the present study developed the following research hypothesis:

H1: Authentic leadership is positively related to individual performance.

### Authentic Leadership, Affective Commitment, and Individual Performance

Authentic leadership has been shown to be an important antecedent of affective commitment (Avolio et al., 2004; Leroy et al., 2012; Rego et al., 2013; Gatling et al., 2016; Semedo et al., 2016, 2019; Delić et al., 2017; Milić et al., 2017; Ribeiro et al., 2020). According to Braun et al. (2013), employees’ identification with and emotional attachment to their leaders increase these followers’ affective commitment to their organization. For example, by providing relational transparency, behaving honestly, and adopting a balanced information processing system, authentic leaders create better quality relationships with their followers, and the latter reciprocate with stronger affective commitment (Paillé, 2009).

Affective commitment is one of the forms organizational commitment can take. Organizational commitment has been given a solid theoretical foundation by Meyer and Allen (1991, 1997) work. This concept can be defined as a psychological force that binds individuals to their organization and shapes their behavior (Meyer and Herscovitch, 2001).

Meyer and Allen (1991, 1997) also developed a well-accepted model that distinguishes between three distinctive forms of commitment: affective, continuance, and normative. These distinct forms of commitment are similar to organizational commitment in that they are a psychological state that explains and characterizes employees’ relationship with their organization and that can have strong implications for workers’ decisions about membership in the organization in question (Meyer and Allen, 1991). Meyer and Allen (1991) further propose that the types of psychological states foreseen for each form of commitment are quite different. Employees with a strong sense of affective commitment will likely remain in their organization because they want to, while workers with more intense continuance commitment will likely stay because they need to avoid the costs of abandonment. Finally, employees with strong normative commitment will likely remain in their organization because they feel they ought to do so out of a sense of obligation. According to Meyer and Allen (1991, 1997), the model predicts that workers can experience all three forms of commitment to various degrees simultaneously—with only one form being dominant—as each type of commitment can appear as a result of different job-related experiences and have contrasting behavioral implications. Thus, individuals may have quite different commitment profiles that can shape their workplace behavior.

While discussing the consequents of all the forms of commitment, Meyer and Allen (1991) quite clearly expect affective commitment—and, to a lesser extent, normative commitment—to be related positively to job performance factors. In contrast, continuance commitment might be unrelated with these factors. As the three types of commitment are expected to have a different relationship with behaviors and performance factors, a separate analysis of each form of commitment can contribute to more accurate behavioral predictions.

Building on Meyer and Allen (1991) model, Herscovitch and Meyer (2002) proposed an extension of the three-component model to adapt it to fit organizational change scenarios (i.e., affective commitment, normative commitment, and continuance commitment to change). Herscovitch and Meyer (2002) approach facilitates the gathering of evidence for how the three forms of commitment to change are connected with different reactions to organizational change. The adapted model reinforces the basic three-component model’s original premises that different but not mutually exclusive types of commitment have separate motivational roots and that each form of commitment is linked to different behaviors and performance factors.

Despite the multidimensional nature of organizational commitment, the present study focused specifically on affective commitment because the literature suggests it has the strongest effect on job performance factors. For instance, committed employees tend to be better at their jobs and more productive (Meyer et al., 2002; Riketta, 2002; Leroy et al., 2012; Ribeiro et al., 2018b). Employees with higher levels of affective organizational commitment are more willing and motivated to contribute significantly to their organization (Rego and Souto, 2004), increasing in- and extra-role performance (Allen and Meyer, 1996; Meyer et al., 2002; Riketta, 2002; Jaramillo et al., 2005; Vandenabeele, 2009). In addition, the current research model proposed that authentic leadership promotes affective commitment, which in turn increases individual performance, as previous research has shown (e.g., Ribeiro et al., 2018b). To take the above findings into account, the following hypothesis was included in the present study:

H2: The relationship between authentic leadership and individual performance is mediated by affective commitment.

### Authentic Leadership, Individual Creativity, and Individual Performance

Empirical research has linked authentic leadership with individual creativity (e.g., Ilies et al., 2005; Walumbwa et al., 2008; Li et al., 2014; Rego et al., 2014; Zhou et al., 2014; Zubair and Kamal, 2015; Semedo et al., 2017, 2018; Ribeiro et al., 2018a; Khan et al., 2019; Zeb et al., 2019). Authentic leaders’ actions are congruent with their words, values, and beliefs, thereby contributing to open, truthful relationships with their followers and promoting work environments in which employees can exchange ideas and share knowledge with each other (Khan et al., 2019). In this positive environment, creativity is fostered and encouraged (Ilies et al., 2005; Rego et al., 2013; Khan et al., 2019). Authentic leadership also stimulates employees’ positive emotions, thereby increasing their creativity (Gavin and Mason, 2004). More specifically, the constructive feedback that characterizes authentic leaders has been shown to enhance creative behavior (Christensen-Salem et al., 2018).

In addition, employee creativity is an important way to improve job-related outcomes (De Stobbeleir et al., 2011). Researchers have documented that creativity enhances workers’ job performance (Amabile, 1996; Madjar et al., 2002; Im and Workman, 2004; Gilson, 2008; Suh et al., 2010; Zhang and Bartol, 2010). Creativity further promotes novelty, usefulness (Oldham and Cummings, 1996; Shalley et al., 2004; George and Zhou, 2007), independence, confidence, and willingness to take risks (Sternberg and Lubart, 1999), making individuals more adaptable and open to new experiences and thus better able to achieve better individual performance. Based on the above results, a third hypothesis was developed for the present research:

H3: The relationship between authentic leadership and individual performance is mediated by individual creativity.

### Authentic Leadership, Affective Commitment, Individual Creativity, and Individual Performance

This study proposed that authentic leadership raises employees’ level of performance by promoting their affective commitment and thus increasing their creativity. Authentic leaders can enhance respect, dignity, integrity, and trust among followers (Bamford et al., 2013), and workers reciprocate by showing more desired behaviors and emotional attachment. When employees’ emotional relationship to their organization is strengthened (Meyer and Allen, 1991), these individuals are more likely to be motivated to make significant contributions to their organization, including presenting new and creative ideas to solve organizational problems (Semedo et al., 2018). Therefore, employees’ affective commitment positively influences their creativity. Workers with higher levels of creativity also have better individual performance due to increased cognition and motivation and more positive behaviors (Luthans et al., 2007).

The current research was based on the assumption that authentic leadership improves employees’ affective commitment, which promotes creativity, which, in turn, enhances individual performance. The relationship between authentic leadership and individual performance can be established through affective commitment and individual creativity, which have been identified as intermediary elements in this psychosocial process. The present study proposed that authentic leadership’s positive impacts foster employees’ affective bonds, which help these workers be creative, so they are continually looking for challenges and striving to meet targets, thereby producing better performance levels. Taking the above findings into account, the final hypothesis was written as follows:

H4: Affective commitment and individual creativity are sequential mediators in the relationship between authentic leadership and individual performance.

## Methodology

### Participants and Procedures

This study has focused on a single hierarchical level and concentrated on the individual unit of measurement and analysis. A cross sectional-survey design was used to collect quantitative data from a sample of employees. The data were drawn from a sampling frame of employees from different sectors to understand more fully how authentic leadership affects individual performance. The survey started with an informed consent section in which the research goals were explained and the collected data’s anonymity and confidentiality were guaranteed. The respondents were asked to answer questions as honestly as possible, and the instructions explicitly stated that items had no right or wrong answers. Instructions were also provided for how to complete the survey to reduce the occurrence of errors. According to Podsakoff et al. (2003), protecting participant anonymity and diminishing evaluation apprehension contributes to reducing response bias, including avoiding lenient, acquiescent, and socially desirable answers. After reading the informed consent section and agreeing voluntarily to participate in the study, the respondents reported their perceptions of authentic leadership, affective commitment, individual performance, and creativity. The last section contained items regarding socio-professional characteristics (e.g., respondents’ age, gender, education, tenure in their organization, and business sector).

The survey was pretested with a sample of 11 employees of a higher education institution to ensure the questionnaire’s contents were clear to respondents. Subsequently, the survey was made available on the same institution’s website and various social media platforms to collect as many completed questionnaires as possible. A minimum of a 6-month tenure in the respondents’ current organization was established as the inclusion criterion.

A non-probabilistic convenience sample of 214 respondents was obtained after the elimination of incomplete surveys and responses from respondents who did not meet the inclusion criteria. G^∗^Power software was used to calculate the sample size based on statistical power (Faul et al., 2009) and to certify the collected sample’s adequacy. A sample size of 148 was recommended to achieve a statistical power of 0.95 in the model testing phase. Since the present study’s sample size exceeded this number, it was deemed sufficiently large enough to test the model.

The respondents had a mean age of 41.48 years (standard deviation [SD] = 10.56; minimum = 21 years; maximum = 65 years), and 71.0% were females. Most respondents have a higher education degree (81.8%), but 14.0% had between 10 and 12 years of education and 4.2% had completed 9 years of education or less. Concerning tenure, the respondents had a mean tenure of 13.05 years in their current organization (SD = 10.39 years; maximum = 38 years). The respondents worked in different sectors, including education (30.8%), commercial services (20.1%), management and economics services (13.1%), human resource management (9.8%), health (8.4%), and other sectors (17.8%). Slightly more than half of the participants worked for a public organization (51.9%).

### Measures

The respondents indicated their level of agreement with each item on a 5-point Likert scale (1 = “Totally disagree” to 5 = “Totally agree”), except for the authentic leadership measure.

#### Predictor Variable: Authentic Leadership

Respondents’ perceptions of their leaders’ behavior were measured using Walumbwa et al. (2008) scale, which comprises 16 items that assess the construct’s four dimensions. The first two dimensions are self-awareness (e.g., “…seeks feedback to improve interactions with others”) and relational transparency (e.g., “…is willing to admit mistakes when they are made”). The third and fourth dimensions are internalized moral perspective (e.g., “…makes decisions based on his/her core beliefs”) and balanced processing (e.g., “…listens carefully to different points of view before coming to conclusions”).

The respondents reported how often their direct supervisors adopted each behavior on a 5-point Likert scale (1 = “Never” to 5 = “Often or always”). To obtain a composite authentic leadership score, the procedure suggested by Luthans et al. (2008) was followed. Thus, the values of the items assessing the four dimensions were first calculated to produce a composite average for each dimension. Then, the averages for the four dimensions were combined to arrive at a composite authentic leadership score for each participant (alpha [α] = 0.94). Higher scores represent stronger perceived authentic leadership.

#### Mediator 1: Affective Commitment

Participants’ affective bonds to their organizations were measured using three items adapted from Rego et al. (2010) (e.g., “I have a strong connection to this organization”). Each respondent’s composite score was calculated by averaging the pertinent items (α = 0.83). Higher scores denote stronger affective commitment.

#### Mediator 2: Individual Creativity

Respondents’ workplace creativity was self-assessed using Zhou and George (2001) scale. The items include descriptions of 13 behaviors (e.g., “I am not afraid to take risks”). Each respondent’s composite score was calculated by averaging all the items (α = 0.92). Higher scores represent stronger perceived individual creativity.

#### Criterion Variable: Individual Performance

Participants’ individual workplace performance was measured using four items developed by Staples et al. (1999) (e.g., “I’m an efficient worker”). Each participant’s composite score was calculated by averaging the items (α = 0.76). Higher scores denote stronger individual performance.

#### Covariate Variables

In line with the existing literature, the present study controlled for some demographic variables. The latter included respondents’ gender, age, and education (e.g., Shalley et al., 2004; Kwan et al., 2018).

### Confirmatory Factor Analyses Testing Discriminant and Convergent Validity

Given that the current research collected data from a single source for all constructs, at a single moment in time, common method variance (CMV) could weaken the results’ validity. CMV refers to spurious covariance between variables resulting from the use of a single data source or method (Podsakoff et al., 2003). To examine whether the four variables’ items capture distinct constructs as opposed to creating common-source bias, confirmatory factor analyses were performed. The four-factor model fit the data well (i.e., root mean square error of approximation [RMSEA] = 0.06; Tucker-Lewis index [TLI] = 0.91; comparative fit index [CFI] = 0.91), while the single-factor model presented unacceptable fit statistics (i.e., RMSEA = 0.14; TLI = 0.47; CFI = 0.50) (Hu and Bentler, 1999; Marsh et al., 2004). In addition, Harman’s single factor technique was also applied. An exploratory factor analysis without rotation was performed, revealing that the first factor accounts for only 29.61% of the total variance (65.04%). These results indicate that the four constructs show discriminant validity and that no serious common-method bias was present in the present study.

Next, all the variables’ composite reliability (CR) and average variance extracted (AVE) were estimated (see Table 1). The CR values (i.e., from 0.77 to 0.99) are well above the recommended cut-off point of 0.70 (Hair et al., 2010). The AVE values estimated for individual creativity (0.49) and individual performance (0.47) are slightly below the widely-accepted threshold of 0.50 proposed by Fornell and Larcker (1981). This suggests that the variance captured by the underlying latent constructs is lower than the variance due to measurement error. As noted by Fornell and Larcker (1981), the AVE is a more conservative estimate of convergent validity than CR and, on the basis of CR alone, the researcher “may conclude that the convergent validity of the construct is adequate, even though more than 50% of the variance is due to error” (p. 46). As the CR values of the constructs are above the recommended level, their convergent validity was considered acceptable to continue with data analysis (for similar decisions see; Lam, 2012; Hardesty et al., 2012; De Nisco et al., 2016; Dijkhuizen et al., 2018). The AVE values were then compared to the squared correlations between all pairs of variables as suggested by Fornell and Larcker (1981) to assess discriminant validity (see Table 1). The comparison revealed that the AVE values are greater than the shared variance between variables—as recommended by Fornell and Larcker (1981) and Hair et al. (2010)—thereby providing some assurance of the indicators’ discriminant validity. According to Valentini and Damásio (2016), fixed cut-off points should be used with caution to avoid limiting the interpretation of empirical research’s results since CR and AVE values can change according to the number of items and factor loadings’ homogeneity. Despite the above-mentioned reservations, overall, the measures were deemed to possess acceptable reliability and validity properties.

**TABLE 1:** Means, SDs, correlations, Cronbach’s α, CRs, and AVEs.

	M	SD	1	2	3	4	5	6	7	CR	AVE
1. Gender^1^	–	–	–	–	–	–	–	–	–	–	–
2. Age	41.48	10.56	–0.08	–	–	–	–	–	–	–	–
3. Education^2^	–	–	0.07	0.21**	–	–	–	–	–	–	–
4. Authentic leadership	3.36	0.91	−0.04	0.13	0.03	(0.94)	0.19	0.03	0.08	0.99	0.97
5. Affective commitment	4.03	0.82	0.05	0.11	0.03	0.44**	(0.83)	0.05	0.28	0.83	0.62
6. Individual creativity	3.84	0.58	−0.21**	0.21**	0.22**	0.17*	0.23**	(0.92)	0.12	0.93	0.49
7. Individual performance	4.06	0.62	−0.06	−0.07	−0.11	0.28**	0.53**	0.34**	(0.76)	0.77	0.47

Spearman’s correlations below the diagonal; squared correlations above the diagonal; * *p* < 0.05; ** *p* < 0.01; ^1^ gender: 0 = male, 1 = female; ^2^ education: 1 = 9 years of education or less, 2 = between 10 and 12 years of education, 3 = higher education degree; Cronbach’s α in parentheses.

## Results

Table 1 presents the means, SDs, and Spearman correlation coefficients. The main variables are all positively and significantly correlated with each other, producing low to moderate correlation coefficients. Gender, age, and education are significantly correlated with individual creativity. However, their correlation coefficients with the remaining variables are statistically non-significant.

PROCESS macro for IBM SPSS version 26 software (Hayes, 2013) was used to evaluate mediation effects. The respondents’ gender, age, and education were set as covariates. Table 2 presents the results for Model 6 obtained through sequential mediation analysis.

**TABLE 2:** Regression coefficients, standard errors, model summary information, and indirect effects for the serial mediator model.

	Affective commitment (mediator 1)		Individual creativity (mediator 2)		Individual performance (criterion variable)	
			
*Total effects*	*B*	SE	*B*	SE	*B*	SE
Constant	–	–	–	–	3.95***	0.29
Authentic leadership	–	–	–	–	0.22***	0.05
Gender^1^	–	–	–	–	−0.05	0.09
Age	–	–	–	–	0.00	0.00
Education^2^	–	–	–	–	−0.18*	0.08
					*F*(4,209) = 7.11; *p* < 0.001; *R*^2^ = 0.12	
***Direct effects***						
Constant	2.78***	0.37	2.51***	0.30	2.19***	0.32
Authentic leadership	0.39***	0.06	0.00	0.04	0.07	0.04
Affective commitment	–	–	0.16***	0.05	0.34***	0.05
Individual creativity	–	–	–	–	0.27***	0.06
Gender^1^	0.11	0.11	−0.31***	0.08	−0.01	0.08
Age	0.01	0.00	0.01*	0.00	−0.01*	0.00
Education^2^	–0.14	0.10	0.21***	0.07	−0.18**	0.07
	*F*(4,209) = 12.96; *p* < 0.001; *R*^2^ = 0.20		*F*(5,209) = 8.86; *p* < 0.001; *R*^2^ = 0.18		*F*(6,209) = 21.44; *p* < 0.001; *R*^2^ = 0.38	

***Indirect effects***	**Effect**		**BootLLCI**		**BootULCI**	

Total	0.15		0.09		0.22	
AL– > AC– > IP	0.13		0.08		0.20	
AL– > IC– > IP	0.00		−0.02		0.03	
AL– > AC– > IC– > IP	0.02		0.00		0.03	

*p < 0.05; **p < 0.01; ***p < 0.001; ^1^ gender: 0 = male, 1 = female; ^2^ education: 1 = 9 years of education or less, 2 = between 10 and 12 years of education, 3 = higher education degree; AL = authentic leadership; AC = affective commitment; IC = individual creativity; IP = individual performance.

The first hypothesis proposed that a positive relationship exists between authentic leadership and individual performance. As shown in Table 2, authentic leadership’s total effect on individual performance is statistically significant (non-standardized coefficient [*B*] = 0.22; *p* < 0.001), indicating that direct supervisors’ adoption of a stronger authentic leadership style increases employees’ workplace performance. Hypothesis H1 thus received empirical support.

The second hypothesis stated that affective commitment mediates the link between authentic leadership and employees’ performance. The results confirm that authentic leadership significantly predicts employees’ affective bond to their organization (*B* = 0.39; *p* < 0.001) and the latter also significantly predicts reported levels of individual performance (*B* = 0.34; *p* < 0.001). In addition, authentic leadership’s indirect effect is statistically significant, which provides evidence of a mediation effect (*B* = 0.13; lower level of confidence interval [LLCI] = 0.08; upper level of confidence interval [ULCI] = 0.20). Hypothesis H2 was, therefore, confirmed.

The third hypothesis posited that individual creativity also has a mediation effect on the relationship between authentic leadership and individual performance. Although the findings indicate that individual creativity helps explain employees’ performance (*B* = 0.27; *p* < 0.001), the level of perceived authentic leadership does not have a significant impact on the respondents’ capacity for providing new useful ideas and solutions in the workplace (*B* = 0.00; non-significant). The indirect effect is not statistically significant (*B* = 0.00; LLCI = –0.02; ULCI = 0.03), thereby verifying that no noteworthy mediation effect exists. Hence, hypothesis H3 did not receive empirical support.

**FIGURE 1:**
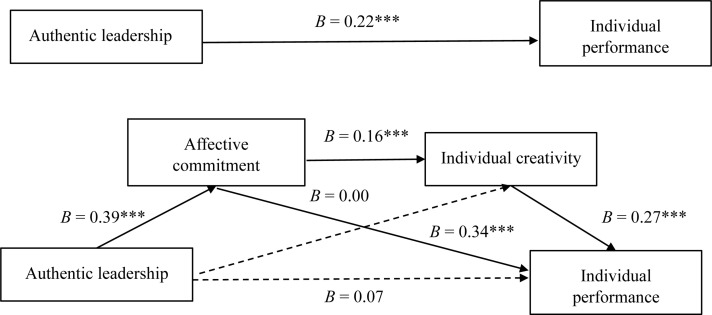
Authentic leadership’s influence on individual performance through affective commitment and individual creativity. ^∗∗∗^*p* < 0.001; *n* = 214; participants’ gender, age, and education function as covariates.

Finally, hypothesis H4 stated that affective commitment and individual creativity serially mediate the relationship between authentic leadership and employees’ performance. The indirect effect of authentic leadership on performance through affective commitment and individual creativity’s mediation is statistically significant (*B* = 0.02; LLCI = 0.00; ULCI = 0.03). Thus, the results show that supervisors’ adoption of authentic leadership behavior is associated with workers’ stronger affective commitment (*B* = 0.39; *p* < 0.001), which then fosters higher levels of individual creativity (*B* = 0.16; *p* < 0.001), which further subsequently contributes to better individual performance (*B* = 0.27; *p* < 0.001). These findings provide support for hypothesis H4, which meant that all the hypotheses could be accepted except for hypothesis H3. The model explains 38% of the unique variance of individual performance (*F*[6, 209] = 21.44; *p* < 0.001). Figure 1 presents the main results.

## Discussion

This study sought to investigate authentic leadership theory—a topic that has already attracted many scholars’ attention. The present research focused on authentic leadership’s impact on employees’ job-related attitudes and behaviors, namely, affective commitment (i.e., attitude), individual creativity, and individual performance (i.e., behavior), as these have important consequences for organizational performance. This study was a response to researchers’ calls for more studies of authentic leadership’s effects (Gardner et al., 2011; Avolio and Walumbwa, 2014; Alilyyani et al., 2018). More specifically, the current investigation analyzed affective commitment and individual creativity’s sequential mediation to understand more fully the psychosocial mechanisms that link authentic leadership to improved employee performance.

The present findings indicate a positive association exists between authentic leadership and workers’ better performance, thereby supporting hypothesis H1. Similar results have been reported by other authors (Clapp-Smith et al., 2009; Wang et al., 2014; Ribeiro et al., 2018a), so the current findings reinforce the existing evidence for a positive relationship between the two constructs. As expected, the relationship between authentic leadership and individual performance is also mediated by affective commitment, which confirms hypothesis H2. The results thus confirm that this significant mediating variable helps explain how authentic leadership promotes higher employee performance (Ribeiro et al., 2018b; Semedo et al., 2016, 2019). According to social learning (Bandura, 1977) and social exchange theories, workers’ behavior is contingent on their leader’s behavior (Cropanzano and Mitchell, 2005). From this perspective, employees engage in positive behaviors when they perceive that their organization is treating them well because they want to reciprocate with better performance. Authentic leadership can be indicative of a tendency toward positive organizational treatment, so this leadership style inspires workers to respond with affective commitment and, consequently, improved performance.

The findings regarding individual creativity’s mediating role in the relationship between authentic leadership and individual performance unexpectedly have no statistically significant mediation effect, leading to the rejection of hypothesis H3. Although one of the conditions for mediation exists (i.e., creativity explains individual performance), authentic leadership apparently does not affect the respondents’ creativity, which prevents the mediation effect from occurring. This result might be explained by the rather weak statistically significant correlation between authentic leadership and creativity in the present sample (*r* = 0.17; *p* < 0.05), which is much lower than that reported in other studies. For example, Ribeiro et al. (2020) correlation values were *r* = 0.64 and *p* < 0.001, while Rego et al. (2012) reported *r* = 0.65 and *p* < 0.001 and Semedo et al. (2016) *r* = 0.46 and *p* < 0.01. Concurrently, the low variability in the present study’s results regarding reported levels of individual creativity (SD = 0.58) may have conditioned the correlations between the variables. From a theoretical standpoint, applying some of social exchange theory’s propositions (Blau, 1964) can be more challenging in the case of creative behavior. For instance, the success proposition states that behavior that generates positive results is likely to be repeated, while the stimulus proposition asserts that behavior rewarded in the past is likely to be repeated in the future. However, workers could regard creative behavior as either stimulated or successful behavior in their workplace. Previous research has found that individual creativity has a significant mediation effect on the relationship between authentic leadership and individual performance (Semedo et al., 2017, 2018), so future studies need to devote further attention to clarify this issue.

In contrast, the last hypothesis received support. The current results offer evidence of the role of employees’ affective commitment and individual creativity as psychosocial sequential mediation mechanisms that explain the relationship between workers’ perceptions of their supervisors’ authentic leadership behavior and these employees’ workplace performance. This finding is innovative, adding to the existing knowledge about the four variables’ interrelationships. The conclusion can be drawn that significant indirect relationships exist between the research model’s main variables via the proposed mediating variables.

As previously mentioned as part of the research background, the above results may be due to how employees’ identification with and attachment to leaders increase these workers’ emotional connection to their organization (Gatling et al., 2016; Delić et al., 2017). This strong link, in turn, stimulates their ability to come up with creative ideas and solutions (Semedo et al., 2018). When employees develop high levels of creativity, as a rule, these workers ultimately produce better results than those who do not have this behavior. That is, creative employees’ better individual performance is due to increased cognition and motivation and more positive behavior (Luthans et al., 2007). Previous research has confirmed each mediating variable’s role individually, but the present study adds to the literature by elucidating their combined and sequential mediation effects.

One especially intriguing result is that the mediation mechanism between authentic leadership, affective commitment, and individual performance is stronger than the sequential mediation effect. This finding could be explained by the aforementioned weak relationship between authentic leadership and creativity in the present sample’s data. Regardless, the results indicate that individuals’ affective bonds to their organization are important as a mechanism by which leaders’ behavior can influence their followers’ outcomes.

### Limitations and Future Research

Despite this research’s valuable contributions, several limitations should be considered when interpreting and generalizing the findings. One limitation is that the study’s design limits the confirmation of any causal nexus amongst the variables. The choice of which direction to take was theoretical driven, but the results’ correlational nature meant causality between variables could not be firmly identified. Future research could focus on longitudinal research design to reach more valid conclusions about causality.

Another limitation is due to the collection of cross-sectional data from a single source, which can lead to spurious covariance among variables. To diminish the possibility of common method bias, the respondents were assured of both their data’s confidentiality and anonymity and the lack of right or wrong answers in order to reduce apprehension about their responses (Podsakoff et al., 2003). Exploratory and confirmatory factor analyses further helped to establish the measures’ discriminant and convergent validity. However, future studies could adopt a two-source method (i.e., surveying both leaders and employees) or a time-lagged data collection strategy to reduce more effectively the potential occurrence of CMV. Regarding convergent validity, individual performance and individual creativity’s measures revealed AVE values a little below the cut-off point of 0.50. Their convergent validity was deemed acceptable to proceed with data analysis based on CR values alone (Fornell and Larcker, 1981). Future research might devote further attention to these measures and introduce potential improvements on the psychometric front. Finally, the data were collected from a non-probabilistic sample, which limits the generalization of results to other organizations.

Despite these limitations, the findings contribute significantly to the literature, especially regarding authentic leadership and individual performance, and open new paths for further research. The present study confirmed that affective commitment is an important mechanism through which leaders can improve their followers’ performance. Future research could adopt a profile approach to investigating organizational commitment (Meyer and Allen, 1991, 1997) and examine whether and how employees’ simultaneous levels of the three forms of organizational commitment affect the leader-worker relationship. Further studies may gain benefits from examining other variables (e.g., value congruence, trust, leader-member exchange, perceived organizational justice, satisfaction with management, job resourcefulness, and happiness at work) that might also explain the link between authentic leadership and individual performance. Value congruence, for example, has been highlighted as a key mechanism through which leaders exert their influence on followers’ attitudes and behaviors because interpersonal and social similarities are conducive to trust (Edwards and Cable, 2009).

Another suggestion would be to analyze moderating variables’ intervention in previously established relationships (e.g., organizational virtuousness, ethical infrastructure, and corporate social performance) since individuals’ behavior is also determined by the context in which it occurs. For instance, the level of organizational ambidexterity and simultaneous use of exploration and exploitation strategies (O’Reilly and Tushman, 2013) might affect the value employees expect from engaging in creativity behaviors as a response to their leader’s behavior. Along the same lines, the present research could also be replicated in other contexts to facilitate data comparisons. The adoption of a qualitative or mixed method approach to examining the relationships under study could further help to deepen the existing understanding of how authentic leadership promotes improved performance. More information on individual creativity’s role in this process might be obtain using these approaches. Another interesting avenue of research to address is using a generational approach to the issues addressed in the present study, more specifically, to assess to what extent authentic leadership can facilitate the attraction and retention of members of more recent generations and stimulate these workers to excel in their jobs.

### Theoretical and Practical Contributions

The results strengthen the existing literature on authentic leadership, affective commitment, individual creativity, and individual performance. The findings reinforce evidence of authentic leadership’s important role in encouraging positive employee behaviors through workers’ stronger affective bonds, as well as stimulating employees’ creativity. This study investigated the sequential process through which both affective commitment and individual creativity transmit authentic leadership’s impact on workers’ performance, thereby extending the literature on authentic leadership in an important novel direction.

The results have managerial implications as they suggest that organizations and administrators need to recognize the importance of betting on a more genuine, transparent, and authentic leadership style. Organizations should commit to selecting authentic leaders. Leaders further need to be open to critical feedback and consider all relevant information before making decisions, as well as being open about their own ideas, feelings, and emotions and being guided by moral values and standards even when under pressure. Training or mentoring programs can also be developed to achieve this end, helping leaders to recognize the benefits of assuming a more authentic leadership style and develop more effectively their competencies in this area. In other words, organizations must invest in developing increasingly authentic leaders and stimulating employees’ deeper emotional connection to their organization and greater workplace creativity, which will ultimately improve workers’ job performance. Leaders’ ability to foster individuals’ deep affective bonds to their organization appears to be especially important to promoting improved performance directly. The above findings provide organizations with guidelines for how leaders can stimulate employees’ unique results and improve their performance, including two sequential mechanisms through which these psychosocial benefits can be enhanced.

## Data Availability Statement

The raw data supporting the conclusions of this article will be made available by the authors, without undue reservation, upon request.

## Ethics Statement

Ethical review and approval was not required for the study on human participants in accordance with the local legislation and institutional requirements. Written informed consent for participation was not required for this study in accordance with the national legislation and the institutional requirements.

## Author Contributions

AD and NR formulated the research design. AD, NR, AS, and DG collected the data. AD conducted the data analyses. All authors contributed to the article and approved the submitted version.

## Conflict of Interest

The authors declare that the research was conducted in the absence of any commercial or financial relationships that could be construed as a potential conflict of interest.
